# Silent suffering: unveiling factors associated with women’s inability to seek help for intimate partner violence in sub-Saharan Africa (SSA)

**DOI:** 10.1186/s12978-023-01651-7

**Published:** 2023-07-26

**Authors:** Kwamena S. Dickson, Ebenezer N. K. Boateng, David Adzrago, Isaac Y. Addo, Evelyn Acquah, Samuel H. Nyarko

**Affiliations:** 1 Department of Population and Health, University of Cape Coast, Cape Coast, Ghana; 2 Department of Geography and Regional Planning, University of Cape Coast, Cape Coast, Ghana; 3 Center for Health Promotion and Prevention Research, School of Public Health, The University of Texas Health Science Center at Houston, Houston, TX USA; 4 Centre for Social Research in Health, The University of New South Wales, Sydney, Australia; 5 Centre for Health Policy and Implementation Research, Institute of Health Research, University of Health, and Allied Sciences, Ho, Ghana; 6 Department of Epidemiology and Biostatistics, University of South Carolina, Columbia, SC USA

**Keywords:** Seeking help, Domestic violence, Social demography, Unreported violence, Family violence

## Abstract

**Background:**

Evidence shows that intimate partner violence (IPV) occurs more frequently in sub-Saharan Africa (SSA) than in other regions of the world. However, limited empirical studies exist on the help-seeking behaviour of women who had experienced IPV in SSA. This study aimed to examine the help-seeking behaviour of women who had experienced IPV in SSA and the factors associated with their inability to seek help after experiencing IPV.

**Methods:**

This is a quantitative study based on data from the latest demographic and health surveys (DHS) of 24 SSA countries. A sample of 53,446 women aged 15–49 years was included in the study. Associations between women’s background characteristics and their help-seeking behaviour after experiencing IPV were examined using proportions and multivariate logistic regression models.

**Results:**

Overall, 60.7% of the sample did not seek help after experiencing IPV. Women's inability to seek help for IPV was highest in Mali (80.4%) and lowest in Tanzania (43.1%). Women’s level of education, wealth status, marital status, age, occupation, and country of residence had significant associations with ‘not seeking help’ for any type of IPV. Those who experienced generational violence (AOR = 1.26, CI = 1.19, 1.33) and those who justified wife-beating (AOR = 1.09, CI = 1.07, 1.15) had higher odds of not seeking help for any type of IPV compared to those who did not experience generational violence or did not justify wife beating. Women who experienced emotional violence (AOR = 0.53, CI = 0.51, 0.55) and physical violence (AOR = 0.74, CI = 0.70, 0.76) had lower odds of not seeking help for any type of IPV compared to their counterparts who did not experience these types of violence.

**Conclusion:**

Women’s inability to seek help for IPV is common in many SSA countries. This study shows that several socio-demographic factors, such as women's age, educational levels, wealth status, and marital status are associated with their inability to seek help for IPV. Additionally, women's justification of wife beating and experience of generational abuse are strongly associated with their inability to seek help for IPV. These factors need to be considered critically in IPV interventions in SSA.

**Supplementary Information:**

The online version contains supplementary material available at 10.1186/s12978-023-01651-7.

## Background

Intimate partner violence (IPV) is a major global public health problem [1]. It refers to the behaviour by an intimate partner or ex-partner that causes physical, sexual, emotional, or psychological harm, including physical aggression, sexual coercion, psychological abuse, and controlling behaviours [4]. Evidence shows that anyone can experience IPV, regardless of location, socio-economic status, religious affiliation, or cultural background [4]. However, women are usually the victims of IPV compared to men [2, 3]. The World Health Organisation [4] estimates that about one-third (30%) of women worldwide have experienced IPV or non-partner sexual violence in their lifetime.

Ample evidence shows that sub-Saharan African (SSA) countries often have an exceptionally high annual incidence of this problem [5–7]. A meta-analysis of demographic and health survey (DHS) data collected between 2008 and 2020 estimated that the median prevalence of emotional, physical, and sexual IPV against women in SSA was 32.5%, ranging from 7.6% in Comoros to 50.9% in Sierra Leone [8]. A similar study of 27 SSA countries has reported that between 2010 and 2019, the proportion of adolescents and young women that experienced IPV ranged from 6.5% in Comoros to 43.3% in Gabon, with a median prevalence of 25.2% [9].

Research shows that tolerance and acceptability of IPV against women in many SSA settings contribute to the high occurrence of the problem in the region [7, 10, 11]. A recent study of 27 SSA countries found that 33% of the men justified at least one form of physical IPV [11]. Based on 2003 DHS data, Antai and Antai [10] also noted that 47% of women in the Niger Delta in Nigeria tolerated IPV. These findings indicate an urgent need to encourage women, especially in SSA, to seek help in the event of impending or occurring violence against them, which would contribute to reducing the problem. While encouraging women, especially those in SSA, to seek violence-related support, two important questions need to be addressed. First, are there adequate support programs to help women who have experienced IPV and those at risk of such violence? Second, given the reported scale of tolerance and acceptability of IPV in some SSA settings, do women in SSA who experience IPV seek any form of support?

In response to the first question, we found studies reporting that there are several existing interventions addressing IPV in SSA [12–15]. For instance, a recent systematic review found existing social empowerment programs, including psychological enhancement support and normative change programs for managing violence symptoms and reducing IPV in SSA [16]. In their evidence review, Anderson et al. [12] also found large multifaceted community-based interventions comprising educational programs aimed at empowering women in SSA against IPV. Regarding the second question, however, we found limited empirical studies on the help-seeking behaviour of women who had experienced IPV in SSA. Documented studies on women’s help-seeking behaviour after experiencing IPV were typically focused on regions other than SSA [17–19]. Therefore, there exists a scarcity of studies that have specifically examined underlying socio-demographic and economic factors associated with the underutilisation of support services by women experiencing intimate partner violence (IPV) in SSA. The available related studies, including Ghose and Yaya [34], Muluneh, Alemu and Meazaw [35], and Tenkorang, Zaami, Kimuna, Owusu and Rohn [36] did not adequately explore the factors that contribute to women’s inability to seek help in SSA. Additionally, these primary investigations have predominantly relied on cross-sectional designs, confined to specific countries within SSA, such as Kenya, Uganda, and Ghana [34–36]. This emphasises the necessity for a more comprehensive regional study that examines the socio-demographic and economic factors associated with women’s limited use of interventions following IPV incidents in SSA. Examining factors associated with women’s inability to seek help for IPV in SSA is important for advancing knowledge, improving intervention strategies, empowering women, and informing policy and advocacy efforts. This study therefore aimed at contributing to the family and violence literature by examining the help-seeking behaviour of women who had experienced IPV in SSA. Specifically, we examined the geographic distribution of IPV in SSA and assessed the help-seeking behaviour of women victims in the region. We also appraised help-seeking behaviour by differences in socio-demographic characteristics and the type of violence. Our findings respond to a growing need and interest for insights into the help-seeking behaviour of women who experience IPV in SSA. The findings may be useful for clinicians, social workers, crime agencies, violence reduction advocates, and governments in SSA.

## Methods

### Data source

The study analysed data from the most recent Demographic and Health Surveys (DHS) between 2010 and 2020 in 24 sub-Saharan African (SSA) countries. Countries were selected based on data availability for the variables of interest. The DHS are nationally representative surveys that select samples of women of reproductive age (15–49 years) using a two-stage stratified cluster sampling methodology. The selection of points or clusters (enumeration areas [EAs]) was the first step. The second stage involved systematically selecting households from each cluster or EA. Interviews were conducted with all women between the ages of 15 and 49 who were “usual” of selected households or “visitors” who slept in the household on the night before the surveys were administered. The DHS is ideal for our study because it collects comprehensive information on various topics, including fertility, family planning, infant and child mortality, maternal (antenatal care, delivery, and postnatal care), child (nutrition, abuse), and IPV. Women who had endured any type of IPV were included in the study. A sample of 53,446 women in total was drawn from 24 countries. The MEASURE DHS approved the use of the data set after reviewing our concept note. The datasets are available to the public for free at The DHS Program—Available Datasets.

### Definition of key violence types

#### Generational violence

In the context of the DHS reports, the concept of generational violence can be understood as the transmission or perpetuation of violence across generations within a family or community. It refers to the phenomenon where individuals who have experienced violence in their childhood or youth are more likely to exhibit or experience violence as adults, and this cycle continues within their own families or communities [37].

#### Emotional violence

The term “emotional violence” is commonly used in the field of public health and in surveys, including the DHS, to describe a form of violence that affects individuals’ emotional well-being and mental health. While the specific definition and terminology may vary across different surveys, including the DHS, emotional violence generally refers to behaviours or actions that are intended to cause emotional distress, humiliation, or psychological harm to an individual [37].

#### Physical violence

In the context of the DHS reports, physical violence refers to a form of violence characterised by the use of physical force or actions that result in physical harm or injury to an individual. It involves acts of physical aggression, such as hitting, slapping, kicking, punching, or any other form of bodily assault, inflicted by one person upon another [37].

#### Sexual violence

In the DHS reports, sexual violence refers to a form of violence that involves non-consensual or forced sexual acts or behaviours perpetrated against an individual. It encompasses a range of acts, including but not limited to forced sexual intercourse, attempted rape, unwanted sexual touching, sexual coercion, or any other form of sexual assault [37].

### Data collection

Demographic and Health Surveys (DHS) are characterised by standardised methods of data collection, ensuring comparability across countries and over time. The data collection process encompasses several approaches employed by DHS surveys. Notably, structured questionnaires are utilised, administered to eligible respondents within selected households, covering a wide range of topics related to population, health, and socio-economic characteristics. The household questionnaire captures information on household composition, socio-economic status, education, and other relevant demographic variables. Concurrently, individual questionnaires are administered to eligible women and men within the selected households, gathering data on various health-related subjects such as intimate partner violence, reproductive health, family planning, maternal and child health, and HIV/AIDS. These questionnaires may also include inquiries regarding education, employment, and other pertinent factors [37]. To complement the questionnaire-based data, DHS surveys also incorporate the collection of biomarker data to objectively assess health conditions. This typically entails the acquisition of blood samples for HIV and anaemia testing, as well as the measurement of height and weight for nutritional assessments [37]. The responsibility for administering the questionnaires and conducting data collection activities lies with trained field teams comprising interviewers and supervisors. These teams undergo standardised training in survey methods, ethical considerations, and interview techniques, ensuring consistency and maintaining quality across all survey sites [37]. Data quality assurance is a key aspect of DHS surveys, employing various strategies throughout the process. These strategies include pre-testing questionnaires prior to the main survey, conducting interviewer training and supervision, implementing data editing and validation procedures, and performing periodic field quality control checks [37]. Following data collection, the gathered information is entered into computer systems using specialised software. A comprehensive data cleaning, validation, and editing process is subsequently carried out to identify and rectify any inconsistencies or errors. Weighting procedures are then applied to account for the complex survey design and non-response bias, enabling population-level estimates [37]. Ethical considerations hold significant importance in DHS surveys, with a strong commitment to obtaining informed consent from participants prior to data collection. The confidentiality and privacy of respondents are rigorously upheld throughout the survey process [37].

### Study variables and measurements

#### Outcome variable

The outcome variable for this study was “not seeking help after experiencing IPV.” The DHS assessed IPV in two different ways. Firstly, they measured the proportion of women who had ever experienced violence. Secondly, they examined those who had experienced violence within the previous 12 months leading up to the survey [37]. In this analysis, we utilised the broader measure, focusing on ever experienced violence when they were in a marital relationship and whether they sought help the last time violence occurred. This variable was derived from the question, “did the respondent seek help from anyone about the IPV?.” The response was captured as “no” and “yes.”

#### Explanatory variables

Following theoretical and empirical literature, twelve explanatory variables were used [11, 20, 21]. The main explanatory variables include generational violence, emotional violence, physical violence, sexual violence, and justification of wife beating.

The generational violence was created in response to questions “ever physically hurt by father” and “ever physically hurt by mother” (no, yes). The experience of any emotional violence by husband/partner, the experience of any physical violence by husband/partner, and the experience of any sexual violence by husband/partner was measured as “yes” and “no”. It was a composite variable derived from reasons due to (1) neglect of a child; (2) burning of food; (3) arguing with husband/partner; (4) refusal to have sex with husband/partner; and (5) going out without permission. These were measured as yes = 1 or no = 0. An index was created with all the “yes” and “no” answers, with scores ranging from 0 to 5. The 0 scores were labelled “no,” and 1 to 5 was labelled “yes.” The Cronbach’s alpha for the data was 0.85. Also, socio-demographic variables such as women’s age, occupation, level of education, wealth status, type of residence, and country of residence were included as confounding variables for the study (see Additional file 1: Table S1).

### Analytical procedure

Descriptive and inferential analyses were carried out. The descriptive analysis looked at the bivariate analysis between the country and outcome variables. It also showed the frequency and proportions of the background characteristics by the outcome variables. A multivariate analysis was utilised using a binary logistic regression model to determine the association between the outcome and explanatory variables. A binary logistic regression model was used based on the dichotomous nature of the outcome variable. Two models were fit. The first model examined the relationship between the main independent variables (generational violence, emotional violence, physical violence, sexual violence, and justified wife beating) and the outcome variable (help-seeking behaviour). The second model examined the relationship between the outcome variable (help-seeking behaviour) and the main explanatory variables (generational violence, emotional violence, physical violence, sexual violence, justified wife beating) after adjusting for the confounding variables (place of residence, level of education, wealth status, women’s age, occupation, and country variable). Each variable was subjected to a multicollinearity test, which revealed a mean–variance inflation factor (VIF) of 3.30 for the variables in the models. According to Midi, Sarkar, and Rana [33], a VIF score higher than 10 indicates the presence of multicollinearity. Adjusted odds ratios with 95% confidence intervals were calculated for each variable. Stata Version 17 was used to analyse the data. To account for any under-or over-sampling in the sample, the results were sample weighted.

## Results

Out of 53,446 women who endured any type of IPV, 60.7% did not seek help. Eight out of ten women from Mali, compared to four out of ten women in Tanzania (43.1%), did not seek help for any type of IPV (Additional file 1: Table S1).

### Background characteristics and the proportion who did not seek help for any type of intimate partner violence

More than half of women who experienced IPV in rural (60.6%) and urban (61.0%) areas did not seek help from anyone. Sixty-eight percent of women with secondary education who experienced IPV did not seek help from anyone compared to 57.7% of women with primary education. A larger proportion of women within the richest wealth status (63.5%) who experienced IPV did not seek help from anyone compared to women with poorer wealth status. Six in ten married women who experienced IPV did not seek help from anyone compared to four in ten divorced women. More women aged 15–19 years (68%) who experienced IPV did not seek help from anyone compared to women aged 40–44 years (60.4%). More unemployed women who experienced IPV (66.0%%) did not seek help from anyone compared to women who were working (59.3%). For violence experience, more than half of women who experienced generational violence (68.1%) did not seek help from anyone. More than half of women who experienced emotional violence (52.2%) did not seek help from anyone. Seven in ten women (70.0%) who experienced physical violence did not seek help from anyone. About 58% of women who had experienced sexual violence did not seek help from anyone. Six in ten women (61.3%) who justified wife-beating did not seek help from anyone compared to those who did not justify wife-beating (Table 1).

**Table 1:** Explanatory variables, the proportion who did not seek help from anyone, and Chi-squared tests

Variable	Frequency (n = 53,446)	Proportion who did not seek help from anyone (%)	χ^2^ (p-value)
*Demographic*			
Residence			χ^2^ = 1.54 (< 0.001)
Urban	20,054	61.0	
Rural	33,392	60.6	
Education			χ^2^ = 162.77 (< 0.001)
No education	16,413	63.7	
Primary	21,046	57.7	
Secondary	14,201	61.0	
Higher	1786	68.5	
Wealth status			χ^2^ = 65.82 (< 0.001)
Poorest	10,626	59.6	
Poorer	11,049	59.0	
Middle	10.714	60.0	
Richer	10,847	61.1	
Richest	10,210	63.5	
Current marital status			χ^2^ = 559.17(< 0.001)
Never in a marital union	406	60.6	
Married	32,572	63.5	
Cohabitation	11,881	60.0	
Widowed	1789	57.9	
Divorced	2050	46.2	
Separated	4748	49.9	
Age (years)			χ^2^ = 81.52 (< 0.001)
15–19	2744	68.0	
20–24	9113	61.6	
25–29	11,481	61.4	
30–34	10,075	59.9	
35–39	8656	59.4	
40–44	6367	58.4	
45–49	5010	60.4	
Occupation			χ^2^ = 169.00 (< 0.001)
Not working	11,201	66.0	
Working	42,245	59.3	
*Violence experience*			
Generational violence			χ^2^ = 232.90 (< 0.001)
No	45,740	59.5	
Yes	7706	68.1	
Emotional violence			χ^2^ = 1.7e + 03 (< 0.001)
No	25,068	70.3	
Yes	28,378	52.2	
Physical violence			χ^2^ = 856.86 (< 0.001)
No	15,650	70.0	
Yes	37,706	56.9	
Sexual violence			χ^2^ = 85.88 (< 0.001)
No	38,699	61.8	
Yes	14,747	57.8	
Justify wife beating			χ^2^ = 5.78 (0.016)
No	25,992	60.0	
Yes	28,254	61.3	
Total	53,446	60.7	

### Likelihood of women not seeking help for any type of intimate partner violence

Our study showed that generational violence, emotional violence, physical violence, justifying wife beating, level of education, wealth status, marital status, age, occupation, and country of residence had a significant relationship with not seeking help for any type of IPV.

A lesser likelihood of not seeking help for IPV was observed among women who experienced emotional violence (AOR = 0.53, CI = 0.51, 0.55) compared to those who did not experience emotional violence. Those who experienced physical violence (AOR = 0.74, CI = 0.70, 0.76) were less likely to not seek help for any type of IPV compared to those who experienced physical violence. Women with secondary education (AOR = 0.87, CI = 0.82, 0.92) had a lesser likelihood of not seeking help for any type of IPV compared to women without education.

A lesser likelihood of not seeking help for any type of IPV was observed among women who were divorced (AOR = 0.54, CI = 0.40, 0.72) compared to those who had never married. Women aged 40–44 years (AOR = 0.81, CI = 0.73, 0.89) were less likely not to seek help for any type of IPV compared to those who were aged 15–19 years. Women who were working (OR = 0.95, CI = 0.92, 0,98) had a lesser likelihood of not seeking help for any type of IPV compared to women who were not working.

Our findings also showed that women who experienced generational violence (AOR = 1.26, CI = 1.19, 1.33) had a higher likelihood of not seeking help for any type of IPV compared to women who did not experience generational violence. Women who justified wife-beating had a higher likelihood of not seeking help for any type of IPV (AOR = 1.09, CI = 1.07, 1.15) compared to those who did not justify wife-beating. Women with the richest wealth status (AOR = 1.14, CI = 1.06, 1.23) were more likely not to seek help for any type of IPV compared to those with the poorest wealth status. A higher likelihood of not seeking help for any type of IPV was observed among women from Mali (AOR = 2.27, CI = 1.92, 2.69) compared to women from Angola (Table 2).

**Table 2:** Multivariate analysis of women who did not seek help for IPV from anyone

Variable	Model 1 Odds ratio (95% confidence interval)	Model 2 Adjusted odds ratio (95% confidence interval)
*Violence experience*		
Generational violence		
No	Ref.	Ref.
Yes	1.26***(1.20, 1.33)	1.26***(1.19, 1.33)
Emotional violence		
No	Ref.	Ref.
Yes	0.53***(0.51, 0.55)	0.53***(0.51, 0.55)
Physical violence		
No	Ref.	Ref.
Yes	0.73***(0.70, 0.76)	0.74***(0.71, 0.77)
Sexual violence		
No	Ref.	Ref.
Yes	0.99(0.95, 1.03)	0.98(0.94, 1.02)
Justify wife beating		
No	Ref.	Ref.
Yes	1.11***(1.07, 1.15)	1.09***(1.05, 1.13)
*Demographic*		
Residence		
Urban		Ref.
Rural		1.02(0.97, 1.07)
Education		
No education		Ref.
Primary		0.85***(0.81, 0.89)
Secondary		0.87***(0.82, 0.92)
Higher		0.94(0.83, 1.07)
Wealth status		
Poorest		Ref.
Poorer		0.96(0.71, 1.25)
Middle		0.99(0.93, 1.04)
Richer		1.05(0.98, 1.11)
Richest		1.14**(1.06, 1.23)
Current marital status		
Never in union		Ref.
Married		0.94(0.71, 1.25)
Cohabitation		0.85(0.64, 1.13)
Widowed		0.83(0.61, 1.12)
Divorced		0.54***(0.40, 0.72)
Separated		0.62**(0.46, 0.83)
Age (years)		
15–19		Ref.
20–24		0.88**(0.80, 0.96)
25–29		0.84***(0.76, 0.92)
30–34		0.82***(0.75. 0.90)
35–39		0.83***(0.76, 0.92)
40–44		0.81***(0.73, 0.89)
45–49		0.84**(0.76, 0.94)
Occupation		
Not working		Ref.
Working		0.83***(0.79, 0.87)
Country		
Angola		Ref.
Burkina Faso		0.53***(0.46, 0.61)
Benin		0.91(0.79, 1.05)
Burundi		0.87*(0.78, 0.97)
Congo DR		0.81***(0.72, 0.90)
Cote d’Ivoire		0.62***(0.55, 0.71)
Cameroon		0.94(0.83, 1.06)
Ethiopia		1.93***(1.64, 2.28)
Gabon		0.59***(0.52, 0.67)
The Gambia		1.16(0.98, 1.38)
Kenya		0.59***(0.52, 0.67)
Liberia		0.53***(0.46, 0.60)
Mali		2.27****(1.92, 2.69)
Malawi		0.83**(0.73, 0.93)
Nigeria		0.99(0.88, 1.13)
Rwanda		0.50***(0.43, 0.59)
Sierra Leone		0.61***(0.54, 0.68)
Chad		0.90(0.79, 1.02)
Togo		0.74***(0.65, 0.84)
Tanzania		0.47***(0.42, 0.52)
Uganda		0.99(0.89, 1.10)
South Africa		0.70***(0.57, 0.85)
Zambia		0.93(0.83, 1.04)
Zimbabwe		0.86*(0.76, 0.97)

*P < 0.05 **p < 0.01 ***p < 0.001 Ref. = Reference category

## Discussion

This study examined the help-seeking behaviour of women who had experienced IPV and the factors associated with the women’s likelihood of ‘not seeking help’ following the violence. The results demonstrated several significant relationships between various factors and the likelihood of not seeking help. By examining the impact of generational violence, emotional violence, physical violence, justification for wife-beating, level of education, wealth status, marital status, age, occupation, and country of residence, we gained valuable insights into the complex dynamics surrounding help-seeking behaviours in cases of IPV in sub-Saharan Africa (SSA).

The findings highlight a concerning pattern of inadequate help-seeking behaviour among women who had experienced IPV in SSA. Specifically, we found that approximately three-fifths of women in SSA (60.7%) did not seek help after experiencing IPV. This prevalence is alarmingly high and signifies a significant burden of unaddressed violence against women in the region. Comparing our findings to global estimates [4], it is noteworthy that the burden of not-seeking help for IPV among women in SSA is twice as high as the global average. This disparity emphasises the urgent need for targeted interventions and strategies to address the issue of poor help-seeking behaviour in SSA. By understanding the underlying factors contributing to this phenomenon, we can develop effective interventions that empower women to seek help in situations of impending or occurring violence.

The implications of our findings for health and wellbeing are profound. Inability to seek help following IPV can have detrimental consequences for women’s physical and mental health. Women who do not access support services may continue to endure violence, leading to prolonged suffering, physical injuries, and psychological trauma [4, 22]. Moreover, the absence of help-seeking can perpetuate a cycle of violence, as women may feel trapped in abusive relationships without the necessary resources and support to break free [4, 22]. Efforts should be directed towards raising awareness about available support services and removing barriers that hinder women from seeking help. This can be achieved through community education programs, targeted campaigns, and the establishment of safe and confidential spaces where women can access assistance without fear of judgment or reprisal.

Unsurprisingly, the SSA women who experienced generational violence or justified wife beating were more likely to not seek help during or after experiencing IPV. Studies show that women who had childhood experiences of violence or grew up in families with violence often become perpetrators or victims of violence later in life, normalising such experiences and not seeking IPV support [4, 23]. Our findings also revealed that women who experienced emotional, physical, or sexual violence were less likely not to seek help for IPV compared to those who experienced generational violence, suggesting that help-seeking for IPV is associated with the type of violence the women experienced. A possible reason could be that while IPV is deemed universally unacceptable and illegal, some IPV may be normalised or based on cultural norms among women in some families and communities in SSA [23]. The findings may also suggest that emotional abuse may have a lesser inhibitory effect on help-seeking behaviour, possibly because it leaves fewer visible marks and may be more difficult to detect or recognise. Similarly, the findings for women who experienced physical or sexual generational violence underscore the severity of these violence types, which may leave visible evidence and lead to a greater perceived need for seeking assistance or intervention.

Differences in the socio-demographic characteristics of the women may explain their help-seeking behaviour for IPV. Consistent with previous studies [24–27], we found that the women’s age, level of education, wealth status, marital status, employment status, and country of residence were associated with not seeking help for IPV. We found that women older than 19 years, compared to their younger counterparts, were likely to seek help for IPV. The higher likelihood of help-seeking behaviour among older women could be due to increased autonomy and confidence with increasing age, coupled with years of experience and knowledge of support sources [25, 28]. Thus, the older women may have gained autonomy due to their age and feel more confident to make their own decisions [21, 23]. Expectedly, we found that divorced or separated women had lower odds of not seeking help for IPV. Considering that IPV often occurs in marriage, cohabitation, or sexual unions, divorced or separated women may be independent and no longer interested in their broken relationships, which can increase their likelihood of seeking IPV support or reporting their ex-partners.

Education plays a significant role in conflict resolution and help-seeking, especially in SSA. Professionalism and communication or cognitive skills with more education positively impact partner support and help-seeking behaviour [25, 29]. Similarly, the women in our study with at least primary or secondary education had lower odds of not seeking help for IPV compared to those without education, implying that help-seeking for IPV has a positive association with education. This also suggests that education may empower women by increasing their knowledge, awareness, and agency to seek support or escape abusive situations. More education improves economic prospects, financial independence, and confidence or freedom to circumvent socio-cultural practices that tend to barricade autonomy and help-seeking behaviour, including support for IPV [25, 30]. In line with this statement, we found that working or employed women were likely to seek help for IPV. However, our findings also showed that being in the richest wealth quintile was associated with a higher likelihood of not seeking help for IPV. One possible implication of this finding is that women in higher wealth quintiles may have gained economic empowerment and decision-making capacity, which could contribute to a reduced prevalence of IPV in their lives. Economic empowerment can provide individuals with resources, options, and the ability to assert their rights, which may lead to a lower incidence of IPV. Therefore, it is plausible that women with higher wealth status are experiencing less IPV and are consequently less likely to seek help. This implication aligns with existing literature that highlights the positive correlation between economic empowerment and reduced IPV [38, 39]. Other previous studies have suggested that economic independence and financial resources can enhance women’s agency, enabling them to negotiate power imbalances within relationships and potentially prevent instances of IPV [21, 28]. However, it is crucial to interpret this implication with caution and consider alternative explanations for the observed association. While economic empowerment may contribute to a lower prevalence of IPV, it is essential to recognise that the relationship between wealth status and help-seeking behaviour for IPV is multifaceted. Other factors, such as cultural norms, social pressures, fear, shame, or lack of awareness about available support services, can also play significant roles in shaping help-seeking behaviours [38].

The existing body of literature suggests that health outcomes and behaviours, including help-seeking for intimate partner violence (IPV), are influenced by geographic inequalities and socio-economic conditions in SSA countries [4, 31, 32]. However, it is important to critically examine the implications of these findings and contextualise them within the scope of this study. We found that help-seeking for IPV among women varied by country, with higher odds of not seeking help observed in only two countries (Ethiopia and Mali) out of the 24 SSA countries included in the study, as compared to Angola. While this finding highlights potential disparities in help-seeking behaviours, it is essential to consider several factors that may contribute to this variation. Firstly, the differences in help-seeking rates could be influenced by variations in cultural norms, social structures, and traditions across the different countries [40]. Thus, attitudes towards IPV and help-seeking can vary significantly based on cultural beliefs, stigmas, and prevailing gender norms within a given society [40]. Therefore, understanding the socio-cultural context of each country is crucial for interpreting these findings accurately. Secondly, it is important to consider the specific socio-economic conditions and geographic inequalities within each country. Factors such as poverty rates, access to healthcare facilities, availability of support services, and literacy levels can significantly impact help-seeking behaviours [38].

Marital status and age were also important factors associated with the likelihood of not seeking help for IPV. Women who were divorced had a lower likelihood of not seeking help compared to those who had never married. This could be attributed to the potential support networks that may be more readily available to divorced women. Thus, divorced women may join support groups specifically tailored for individuals going through a divorce [41]. These groups offer a safe space for sharing experiences, exchanging advice, and receiving emotional support from others who have faced or are currently facing similar challenges. Support groups can provide validation, reduce feelings of stigma, and enhance coping strategies [41].

Additionally, older women (40–44 years) were less likely not to seek help compared to younger women (15–19 years). With age and life experience, older women may become more assertive and confident in expressing their needs and seeking help when necessary. They may have developed better communication skills and a stronger sense of self-advocacy, enabling them to reach out for support. Older women may have attained a higher level of independence. This increased independence could empower them to seek help without hesitation or reliance on others for assistance. Over time, older women may have accumulated more social, financial, and professional resources. These resources can provide them with a wider range of options and support networks to turn to when facing challenges [42]. Having access to these resources can make help-seeking feel more accessible and achievable.

As our results suggest, women with high socio-economic status were less likely not to seek help for IPV, and the opposite was found to be true for those with low socio-economic status. The higher odds of not seeking help for IPV among the women in Ethiopia and Mali could be due to poor socio-economic conditions leading to limited women empowerment and freedom. However, a further geospatial analysis should be conducted to delineate the geographic inequalities and socio-economic differences within the various countries affecting the women’s ability to seek help for IPV.

### Limitations of the study

While our study has yielded significant findings with implications for researchers and policymakers, it is important to acknowledge several limitations that should be considered. First, due to the cross-sectional nature of our study, we were unable to examine the temporal sequence of help-seeking for intimate partner violence (IPV) and its associated risk factors, which limits our ability to draw causal inferences. Future longitudinal studies are needed to explore the dynamic nature of help-seeking behaviours over time. Second, the measures used in our study, except for the country measure, relied on self-reported data provided by the women themselves. Consequently, recall bias and social desirability biases may have influenced our findings, potentially impacting the accuracy and reliability of the reported information. Third, our analysis was restricted to women aged 15–49 years due to the limitations of the available data from the Demographic and Health Surveys (DHS). This age limitation may not fully capture the experiences of older women or younger adolescents who are also vulnerable to IPV. Future research should aim to include a broader age range to obtain a more comprehensive understanding of help-seeking patterns across different age groups.

Furthermore, our use of the “ever-experienced” IPV measure as a benchmark may oversimplify the complex effects of frequency and severity of violence experienced by women. Future studies could explore more nuanced measures of IPV to better capture the diverse experiences and their implications for help-seeking behaviours. Additionally, our choice of using never married participants as a reference category may introduce potential unreliability compared to women who have been ever married or are in recognised sexual unions. Future studies should consider alternative reference categories that more accurately reflect the diversity of marital or relationship statuses. Furthermore, our study did not investigate geospatial differences in socio-economic conditions and their influence on help-seeking for IPV among women in sub-Saharan Africa. Future research should incorporate spatial analysis techniques to examine how regional variations in socio-economic factors may impact help-seeking behaviours. Lastly, it is important to note that the data used in our study were collected at different time points, spanning from 2010 to 2020. This variability in survey years introduces the possibility of time variations that may have influenced our findings. Care should be taken when interpreting the results in light of these temporal variations. Despite these limitations, our study contributes valuable insights into the field of IPV and help-seeking behaviours in sub-Saharan Africa. Addressing these limitations through further research will enhance our understanding of the complex dynamics involved and guide the development of effective interventions and policies.

## Conclusions

In conclusion, our research findings shed light on the significant issue of help-seeking behaviours among female victims of intimate partner violence (IPV) in sub-Saharan Africa (SSA). Our study reveals that a substantial proportion of women in SSA who experience IPV do not seek help from anyone, with significant variation observed among individual countries. We identified several key factors associated with the likelihood of not seeking help for IPV. Women who had experienced generational violence and those who justified wife beating were more inclined to refrain from seeking help during or after their violent experiences. On the other hand, women who suffered from physical and emotional IPV were less likely to avoid seeking help when compared to their counterparts who did not experience these specific types of IPV. The findings highlight the importance of further research focusing on high-risk groups, specifically generational IPV victims and women who justify wife beating. Understanding the contextual factors underlying their inability to seek help for their IPV experiences is crucial. By delving deeper into the complexities surrounding these groups, we can gain insights that will inform the development of targeted interventions and support services to address their unique needs. Efforts should be made to expand our understanding of the barriers and challenges faced by these high-risk groups, as well as the cultural and social dynamics that influence help-seeking behaviours in SSA. Such research can contribute to the development of effective strategies aimed at empowering and supporting these individuals, ultimately reducing the prevalence and impact of IPV in SSA.

## Supplementary Information

**Additional file 1: Table S1.** Country, survey year, frequency, and the proportion who did not seek help from anyone.

## Data Availability

Datasets are available through the corresponding author upon reasonable request.
