# Supplementary material for: Silent suffering: unveiling factors associated with women’s inability to seek help for intimate partner violence in sub-Saharan Africa (SSA)
Source: Reprod Health. 2023 Jul 26;20:110. doi: 10.1186/s12978-023-01651-7 (PMC10373244; doi:10.1186/s12978-023-01651-7)
Supplement: Supplementary file 1 — Additional file 1: Table S1. Country, survey year, frequency, and the proportion who did not seek help from anyone. [file 12978_2023_1651_MOESM1_ESM.docx]

Table A1: Country, survey year, frequency, and the proportion who did not seek help from anyone

| **Variable** | **Survey Year** | **Frequency (n=53,446)** | **Proportion who did not seek help from anyone (%)** |
| --- | --- | --- | --- |
| Angola | 2015-2016 | 3,520 | 61.9 |
| Burkina Faso | 2012 | 1,849 | 58.7 |
| Benin | 2017-2018 | 1,349 | 64.9 |
| Burundi | 2010 | 3,706 | 63.1 |
| *Congo DR* | 2013-2014 | 3191 | 63.0 |
| Cote D’Ivoire | 2011-2012 | 1,644 | 58.7 |
|  |  |  |  |
| Cameroon | 2018 | 2,082 | 61.8 |
| Ethiopia | 2016 | 1,468 | 78.2 |
| Gabon | 2012 | 2,173 | 54.9 |
| The Gambia | 2019-2020 | 944 | 73.0 |
| Kenya | 2014 | 2,085 | 52.1 |
| Liberia | 2019-2020 | 1,283 | 54.7 |
| Mali | 2018 | 1,556 | 80.4 |
| Malawi | 2015-2016 | 2,377 | 60.5 |
| Nigeria | 2018 | 2,275 | 66.2 |
| Rwanda | 2014 | 865 | 46.9 |
| Sierra Leone | 2019 | 2,504 | 55.7 |
| Chad | 2014-2015 | 2,151 | 64.4 |
| Togo | 2013-2014 | 1,754 | 57.9 |
| Tanzania | 2015-2016 | 3,712 | 43.1 |
| Uganda | 2016 | 4,374 | 65.1 |
| South Africa | 2016 | 913 | 58.1 |
| Zambia | 2018 | 3,208 | 63.1 |
| Zimbabwe | 2015 | 2,466 | 60.7 |
| Total |  | 53,446 | 60.7 |
